# Telemonitoring of Active Inflammatory Bowel Disease Using the App TECCU: Short-Term Results of a Multicenter Trial of GETECCU

**DOI:** 10.2196/60966

**Published:** 2024-11-18

**Authors:** Mariam Aguas, Javier Del Hoyo, Raquel Vicente, Manuel Barreiro-de Acosta, Luigi Melcarne, Alejandro Hernandez-Camba, Lucía Madero, María Teresa Arroyo, Beatriz Sicilia, María Chaparro, María Dolores Martin-Arranz, Ramón Pajares, Francisco Mesonero, Miriam Mañosa, Pilar Martinez, Silvia Chacón, Joan Tosca, Sandra Marín, Luciano Sanroman, Marta Calvo, David Monfort, Empar Saiz, Yamile Zabana, Ivan Guerra, Pilar Varela, Virginia Baydal, Raquel Faubel, Pilar Corsino, Sol Porto-Silva, Eduard Brunet, Melodi González, Ana Gutiérrez, Pilar Nos

**Affiliations:** 1 Gastroenterology Department La Fe University and Polytechnic Hospital Valencia Spain; 2 Health Research Institute La Fe Valencia Spain; 3 Miguel Servet University Hospital Zaragoza Spain; 4 University Clinical Hospital Santiago Spain; 5 Parc Taulí Hospital Universitari Institut d’Investigació i Innovació Parc Taulí (I3PT-CERCA) Sabadell Spain; 6 Nuestra Señora de la Candelaria University Hospital Tenerife Spain; 7 Dr Balmis General University Hospital ISABIAL Alicante Spain; 8 CIBERehd Instituto de Salud Carlos III Madrid Spain; 9 Lozano Blesa Clinic University Hospital Zaragoza Spain; 10 Burgos University Hospital Burgos Spain; 11 Hospital Universitario de La Princesa Instituto de Investigación Sanitaria Princesa (IIS-Princesa) Universidad Autónoma de Madrid Madrid Spain; 12 La Paz University Hospital Faculty of Medicine Universidad Autónoma de Madrid Madrid Spain; 13 Instituto de Investigación Sanitaria del Hospital Universitario La Paz (IdiPAZ) Madrid Spain; 14 Infanta Sofía University Hospital Madrid Spain; 15 Ramón y Cajal University Hospital Madrid Spain; 16 Germans Trias i Pujol University Hospital Badalona Spain; 17 San Cecilio Clinic University Hospital Parque Tecnológico de la Salud Granada Spain; 18 Morales Meseguer General University Hospital Murcia Spain; 19 Clinic University Hospital Valencia Spain; 20 Reina Sofía University Hospital Córdoba Spain; 21 Hospital Alvaro Cunqueiro Vigo Spain; 22 Puerta de Hierro University Hospital Madrid Spain; 23 Consorci Sanitari Terrasa Barcelona Spain; 24 Xarxa Assistencial University Hospital Manresa Spain; 25 Mútua Terrassa University Hospital Terrassa Spain; 26 Fuenlabrada University Hospital Madrid Spain; 27 Cabueñes Universitary Hospital Gijón Spain; 28 Joint Research Unit in ICT Applied to Reengineering Socio-Sanitary Process IIS La Fe-Universitat Politècnica de València Valencia Spain; 29 Physiotherapy in Motion Multispeciality Research Group (PTinMOTION) Department of Physiotherapy Universitat de València Valencia Spain

**Keywords:** clinical trial, telemonitoring, inflammatory bowel disease, Crohn disease, ulcerative colitis, quality of life, socioeconomical and psychological end points, health outcomes, remission time

## Abstract

**Background:**

Telemonitoring for inflammatory bowel disease (IBD) has not consistently demonstrated superiority over standard care; however, noninferiority may be an acceptable outcome if remote care proves to be more efficient.

**Objective:**

This study aims to compare the remission time and quality of life of patients with active IBD managed through standard care versus the TECCU (Telemonitoring of Crohn Disease and Ulcerative Colitis) app.

**Methods:**

A 2-arm, randomized, multicenter trial with a noninferiority design was conducted across 24 hospitals in Spain. The study included adult patients with IBD who were starting immunosuppressive or biological therapy. Participants were randomized into 2 groups: the telemonitoring group (G_TECCU) and the standard care group (G_Control). The follow-up schedule for the telemonitoring group (G_TECCU) was based on contacts via the TECCU app, while the control group (G_Control) adhered to standard clinical practice, which included in-person visits and telephone calls. In both groups, treatment adjustments were made based on the progression of disease activity and medication adherence, assessed using specific indices and biological markers at each check-up. The primary outcome was the duration of remission after 12 weeks, while secondary outcomes included quality of life, medication adherence, adverse events, and patient satisfaction.

**Results:**

Of the 169 patients enrolled, 158 were randomized and 150 were analyzed per protocol: telemonitoring (n=71) and control (n=79). After 12 weeks, the time in clinical remission was not inferior in the telemonitoring group (mean 4.20, SD 3.73 weeks) compared with the control group (mean 4.32, SD 3.28 weeks), with a mean difference between arms of –0.12 weeks (95% CI –1.25 to 1.01; noninferiority *P*=.02). The mean reduction in C-reactive protein values was –15.40 mg/L (SD 90.15 mg/L; *P*=.19) in the G_TECCU group and –13.16 mg/L (SD 54.61 mg/L; *P*=.05) in the G_Control group, with no significant differences between the 2 arms (*P*=.73). Similarly, the mean improvement in fecal calprotectin levels was 832.3 mg/L (SD 1825.0 mg/L; *P*=.003) in the G_TECCU group and 1073.5 mg/L (SD 3105.7 mg/L; *P*=.03) in the G_Control group; however, the differences were not statistically significant (*P*=.96). Quality of life improved in both groups, with a mean increase in the 9-item Inflammatory Bowel Disease Questionnaire score of 13.44 points (SD 19.1 points; *P*<.001) in the G_TECCU group and 18.23 points (SD 22.9 points; *P*=.001) in the G_Control group. Additionally, the proportion of patients who adhered to their medication significantly increased from 35% (25/71) to 68% (48/71) in the G_TECCU group (*P*=.001) and from 46% (36/79) to 73% (58/79) in the G_Control group (*P*=.001). The satisfaction rate remained stable at around 90%, although noninferiority was not demonstrated for the secondary outcomes.

**Conclusions:**

Telemonitoring patients with active IBD is not inferior to standard care for achieving and maintaining short-term remission. The TECCU app may serve as a viable alternative follow-up tool, pending confirmation of improved health outcomes and cost-effectiveness over the long-term.

**Trial Registration:**

ClinicalTrials.gov NCT06031038; https://clinicaltrials.gov/ct2/show/NCT06031038

**International Registered Report Identifier (IRRID):**

RR2-10.2196/resprot.9639

## Introduction

Inflammatory bowel disease (IBD) primarily consists of Crohn disease (CD) and ulcerative colitis (UC), both of which are chronic, relapsing conditions characterized by inflammation of the gastrointestinal tract. Because of its chronic nature, IBD requires continuous and personalized monitoring to prevent medium- and long-term complications. Therefore, effective monitoring strategies must be implemented throughout the disease course to optimize the management of patients with IBD.

Unlike other chronic conditions, IBD primarily affects young individuals during their prime period of personal development. Consequently, IBD is associated with high levels of school and work absenteeism, varying degrees of disability [1], interference with social activities, and a reduced health-related quality of life (HRQoL) [2-4]. In addition, patients with IBD place significantly greater demands on health care resources compared to those with other conditions [2]. As a result, IBD has a considerable medical, social, and economic impact, further amplified by the global increase in its incidence and prevalence in recent years [3,4]. In this context, the most recent data available for Spain show an overall IBD incidence of 16 cases per 100,000 persons per year [5].

To address the challenges in managing these patients, telemedicine applications have been developed to enhance adherence and improve clinical outcomes [6]. Telemonitoring is the primary form of telemedicine used in IBD, concentrating on the structured and continuous monitoring of clinical data that patients self-report from their usual environment. In recent years, web-based telemonitoring systems have been developed, including mobile health (mHealth) tools, which are more cost-effective than home-automated telemanagement programs [7]. Web-based telemonitoring systems are safe, feasible, and cost-effective solutions for patients with IBD [8-12]. Furthermore, their use leads to fewer outpatient visits and hospital admissions [8,9,11,13-17], resulting in reduced health care costs [8,13,14].

Telemonitoring can, in fact, address many aspects of the STRIDE-II strategy for the early detection of potential complications in IBD [18]. Symptomatic responses and remission can be assessed using validated patient-reported outcome measures. Additionally, point-of-care tests now enable the measurement of fecal calprotectin (FC) near the patient. However, to date, telemonitoring has not demonstrated superiority over standard care in terms of health outcome improvements [8,10,11,13,15,19,20], and inconsistent results have been observed across different populations [7].

Even so, noninferiority can be considered an advance if the intervention provides other benefits, such as the cost reductions previously reported with telemonitoring [8,10,11,13,14,17,21,22]. In this context, we have developed a web-based telemanagement system called TECCU (*Telemonitorización de la Enfermedad de Crohn y Colitis Ulcerosa,* or Telemonitoring of Crohn Disease and Ulcerative Colitis) for the remote monitoring of patients with moderate-to-severe IBD who are starting treatment with immunosuppressants or biological agents [23]. In a pilot randomized trial, TECCU was shown to be a safe method for improving disease outcomes, with a more cost-effective profile than standard care, though the improvement in disease control was not statistically superior [20,24,25].

Therefore, telemonitoring has not been demonstrated to be either superior or noninferior to standard care in improving health outcomes, and this lack of data complicates decision-making when considering investments in mature telemedicine programs. As a result, telemonitoring has not been implemented in a structured manner in daily practice, and the reproducibility of results achieved so far has been limited to a few centers. Given these considerations, we adopted a novel noninferiority design for a multicenter trial conducted on a nationwide scale in Spain. This study aims to evaluate the time in remission and QoL of patients with IBD with moderate-to-severe activity managed through telemonitoring (G_TECCU) compared with standard care (G_Control) after 12 weeks.

## Methods

### Study Design

**Figure 1:**
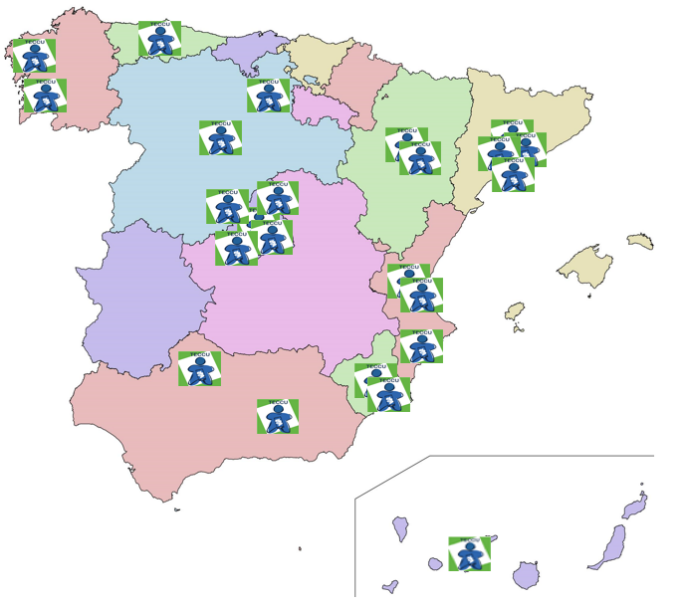
Geographical locations of the participating centers. TECCU: Telemonitorización de la Enfermedad de Crohn y Colitis Ulcerosa or Telemonitoring of Crohn’s Disease and Ulcerative Colitis.

A randomized, open, multicenter, noninferiority trial was conducted across 24 hospitals in Spain. The country has 17 autonomous communities, and patients from 10 of these regions participated in the study, as illustrated in the map in Figure 1. The project was promoted by GETECCU, and the study design was discussed in successive research meetings before final protocol approval. The trial is registered on ClinicalTrials.gov with the identifier NCT06031038.

### Patient Selection and Recruitment

#### Data Collection

Patients were recruited at the 24 participating hospitals between September 2020 and April 2023. The collected data were assessed, stored in a database, and made available for interim analysis starting March 19, 2024 [26,27]. Patients were included consecutively from the outpatient clinics of the IBD Units or the Gastroenterology wards. The inclusion and exclusion criteria (see below) were verified at each visit.

#### Inclusion and Exclusion Criteria

The inclusion criteria were as follows: patients aged 18 years or older diagnosed with CD or UC according to internationally accepted criteria [26,27] and initiation of therapy with immunosuppressants or biological agents (with or without steroids) or both due to disease activity occurring no more than 1 week before inclusion in the study. Active disease at the time of inclusion was defined using clinical indices, biological markers, or endoscopic activity: Harvey-Bradshaw Index (HBI) >4 for CD or Simple Clinical Colitis Activity Index (SCCAI) >2 for UC; or FC values ≥200 μg/g; and/or moderate to severe endoscopic activity (ulcers in CD/endoscopic Mayo Index ≥2 for UC).

The exclusion criteria were as follows: cognitive or sensory impairment; inability to speak or read Spanish without a legally authorized representative capable of participating in the study; transient patients; inability to manage a smartphone, tablet, or computer; lack of a telephone line; participation in other clinical trials during the inclusion period; uncontrolled medical or psychiatric conditions; presence of ileorectal or ileal pouch-anal anastomosis; recipients of a definitive ileostomy; perianal disease; patients with cancer undergoing active treatment; terminal patients or individuals receiving palliative care as defined by the Spanish Society of Palliative Care; institutionalized patients; patients or first-degree relatives who are part of the research team or staff members of the research or health centers participating in the study; and patients undergoing specific follow-up in other units (eg, hemodialysis, transplants) requiring mandatory hospital visits at least every 2 months.

#### Recruitment Process

Patients underwent a face-to-face interview with research staff involved in the study during outpatient visits to the IBD units or during hospitalization for an IBD flare-up. These interviews provided information about the care program and the study, and they were used to obtain written informed consent before inclusion in the study. For patients who agreed to participate, baseline data were collected using a notebook specifically designed for this purpose. Additionally, data regarding biological parameters were obtained from blood and stool samples collected at the initial study visit. If the same biological parameters had been obtained in standard clinical practice less than 2 weeks before enrollment in the study, they were accepted as baseline values.

After a 12-week follow-up, coinciding with the patient’s visit to the IBD unit as part of their routine health care, a face-to-face interview was conducted to complete the case report and collect data on the following: clinical activity indices, QoL using the 9-item Inflammatory Bowel Disease Questionnaire (IBDQ-9), patient satisfaction with treatment, activity at work and productivity, medication adherence, and adverse events (AEs). Additionally, biological parameters from blood and stool samples were measured again during this visit.

#### Randomization

Eligible patients were randomized in a 1:1 ratio into 2 groups: 1 group underwent remote monitoring through TECCU (G_TECCU), while the other group received standard care, which consisted of in-person outpatient visits to the IBD units combined with telephone calls made by physicians or specialized nurses, following standard clinical practice (G_Control). The allocation schedule was based on computer-generated random numbers with a block size of 4 patients. Allocation concealment was ensured using a web-based open-source application for randomization in clinical trials to generate a random allocation sequence. Once a number was assigned, it could not be reassigned, and the members of the research team who were in contact with the patients did not have access to the randomization tables or lists.

The follow-up schedule was the same for both groups, consisting of an in-person visit at baseline and at 12 weeks. The differences between the groups lie in the intermediate controls: in-person visits or telephone calls for G_Control and the TECCU app for G_TECCU. Moreover, additional clinical visits and telephone calls were made at the discretion of the health care providers in both arms if necessary, based on the patient’s clinical evolution. Neither the patients nor the researchers were masked to the intervention; however, the results were analyzed by an independent statistician who was blinded to the group identification.

### Interventions

#### TECCU Telemonitoring App

For G_TECCU, monitoring and management were conducted remotely through the updated TECCU management platform, which was set up according to the patient’s preferences gathered from a series of focus groups [28]. This platform addresses the needs of patients, professionals, and the organization to ensure the efficient provision of health services. It is a multiplatform and flexible solution that supports clinical decision-making and can be integrated with electronic medical records. It is configurable, allowing plans and thresholds to be customized for specific patient profiles. The platform also enables the creation of alerts using different variables, facilitating a holistic approach to patient care.

TECCU operates through a secure web page with an HTTPS app for mobile phones and tablets. During telemonitoring, patients connect to the platform via the app using their personal code and respond to various questionnaires about their disease in the form of chat messages. The questions pertain to the variables used to assess disease status at each check-up, in accordance with a preestablished schedule. Furthermore, the number of check-ups can be increased if necessary to adequately monitor disease evolution during follow-up.

In addition, patients received advice, reminders, and educational materials about their disease and its prevention. The specialized health care personnel at each IBD unit received information from the patients, which was filtered through an intelligent prioritization system to generate alerts based on an integrated intervention protocol. Upon receiving an alert, the health care personnel implemented action plans in accordance with the established intervention protocol to adjust medication and follow-up schedules as needed. These alerts were triggered based on responses to questions regarding activity indices, AEs, and fecal or blood results. Treatments were adjusted using the platform’s messaging system, along with telephone calls or in-person visits when patients required training on the administration of new medications.

#### Standard Care Provided by the IBD Units

The G_Control patients received the usual care provided by the IBD Units (outpatient clinic) for those with moderate-to-severe disease activity, based on national and European clinical guidelines [26,27,29]. Treatment was adjusted according to the evolution of disease activity and medication adherence, measured through specific indices and biological markers during visits or via telephone calls for issues that could be monitored during intermediate check-ups. Additionally, the time in remission was assessed weekly throughout the follow-up based on the patient’s self-recorded clinical activity in a home diary.

This care was complemented by ad hoc hospital care in cases of IBD flare-ups. In such instances, intensive care was maintained until the patient’s condition stabilized, after which they returned to a follow-up regimen based on standard care through the IBD unit. Patients in both arms were provided with all educational materials about IBD available for the remote monitoring of patients. Questionnaires on HRQoL, satisfaction, and work productivity were completed at baseline and again at 12 weeks.

### Study Outcomes

#### Overview

The variables measured at baseline included sociodemographic information, smoking status, disease profile and activity, treatment received, HRQoL, work productivity, impairment in daily activities, medication adherence, and patient satisfaction.

#### Primary Outcome

The primary outcome of the study was to determine the time in remission after 12 weeks of follow-up. This was evaluated by assessing disease activity at baseline and at each check-up during the 12-week follow-up period established for this analysis. Clinical disease activity was evaluated using the modified HBI for patients with CD [30] and the SCCAI, also known as the Walmsley Index, for patients with UC [31]. Clinical remission was defined as an HBI of ≤4 for patients with CD or an SCCAI of ≤2 for patients with UC.

Biological markers were measured at baseline and 12 weeks after inclusion. The laboratory parameters were C-reactive protein (CRP) levels (mg/L) and FC levels (μg/g). Changes in medication were made based on these markers and clinical disease activity indices, following specific intervention plans.

#### Secondary Outcomes

The HRQoL of patients was evaluated at baseline and week 12 using the specific IBDQ-9, a validated tool consisting of 9 items across 4 dimensions: bowel symptoms, systemic symptoms, emotional status, and social behavior. Each item is scored on a 7-point Likert scale, yielding an overall score that ranges from 7 (lowest QoL) to 63 (highest QoL), which is then calculated as a percentage of the maximum score.

The impact of the disease on work productivity and daily activities was assessed at baseline and at week 12 using the Work Productivity and Activity Impairment (WPAI) questionnaire [32]. This questionnaire consists of 6 items that evaluate the disease’s effect on work and daily activities over the past 7 days. The WPAI generates 4 scores expressed as “impairment percentages,” with higher scores indicating a greater impact. The Spanish version has been validated and demonstrated reproducibility in patients with CD.

Medication adherence was evaluated using the 8-item Morisky Medication Adherence Scale (MMAS-8) [33,34], which has been utilized in clinical trials involving patients with IBD [19]. Patient satisfaction with the care received was assessed using an adapted version of the Client Satisfaction Questionnaire. In addition, patient-reported outcomes concerning health status, the presence of abdominal pain, stool frequency, and blood in the stool were also recorded. The safety of the interventions was evaluated by measuring the number of emergency department visits, unscheduled outpatient visits, hospitalizations, and AEs related to medication use.

### Statistical Analysis

As this is a noninferiority study, we conducted a per-protocol analysis. The characteristics of the participants were described using the mean, SD, 95% CI, median, IQR, and range for quantitative variables, as well as absolute and relative frequencies for qualitative variables. Possible baseline differences between the 2 study groups were compared using the Student *t* test for independent samples or the Mann-Whitney *U* test for quantitative variables, and the chi-square test or Fisher exact test for qualitative variables. Time in clinical remission was summarized using the mean, SD, median, IQR, and range.

The sample size was estimated based on the results of the pilot trial, which indicated that patients in the control group remained in remission for a median of 14.3 weeks, whereas those in the G_TECCU group had a median remission time of 17.9 weeks. These differences in time to remission were evaluated with a noninferiority limit of –1.5 weeks, considering this difference to be clinically irrelevant within the 12-week follow-up period of this interim analysis. Noninferiority was assessed by calculating the mean and 95% CI for the difference in the variable of interest between the groups. This was done by comparing the limits of the CI with the predefined noninferiority margin using a Student *t* test. Accepting an α risk of .025 in a 1-sided test, a β risk of .2, and an SD of 3.2, it was determined that 71 patients were needed in each group. Furthermore, assuming a loss rate of 15%, a total of 168 patients was required for the study.

Changes in secondary outcomes from baseline were described by applying appropriate estimators based on the type of variables, as previously outlined. Possible differences in secondary outcomes between the 2 groups were compared using the Student *t* test for independent samples, the Mann-Whitney *U* test for quantitative variables, and the chi-square or Fisher exact test for qualitative variables. All analyses were conducted using SAS statistical software version 9.4 (SAS Institute), with a 1-sided significance level of .025 accepted for all analyses.

### Ethical Considerations

The study protocol was approved by the local independent ethics committee at La Fe University and Polytechnic Hospital, Valencia (v0.3, 14/07/2020), and by the Spanish Agency of Medicines and Medical Devices (AEMPS/Agencia Española de Medicamentos y Productos Sanitarios: 30/07/2020). The study was conducted in accordance with the following: the “Note for Guidance on Good Clinical Practice” (CPMP/ICH/135/95, May 1, 1996); Royal Decree 223/2004 (February 2004); the Helsinki Declaration on ethical principles for medical research involving human subjects, as adopted by the General Assembly of the World Medical Association (7th revised version, Seoul, 2008); guidelines from the International Conference on Harmonization; and the official regulations imposed by the participating centers.

## Results

### Study Sample and Baseline Characteristics

**Figure 2:**
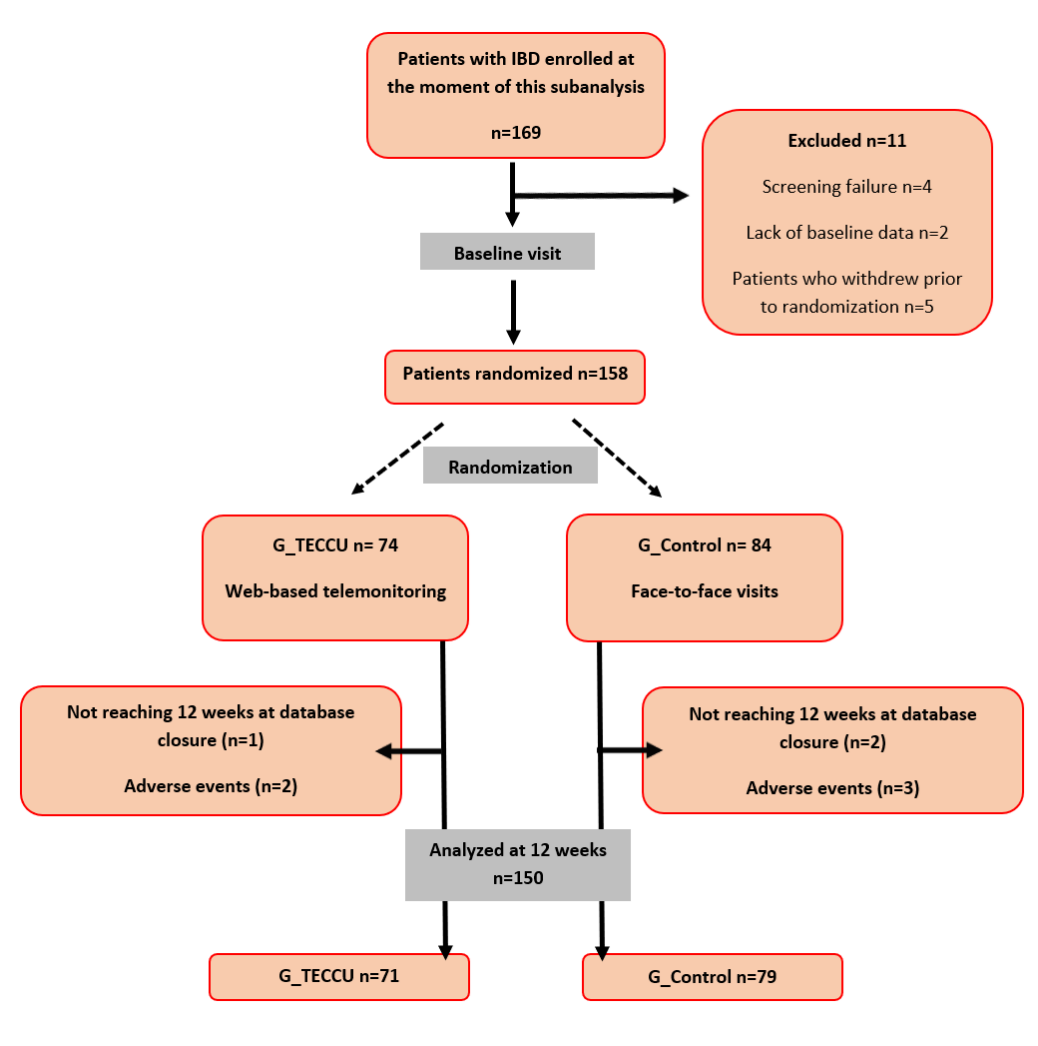
Study flowchart. G_CONTROL is the group receiving standard care with in-person visits and telephone calls as usual practice; G_TECCU is the group receiving remote monitoring. IBD: inflammatory bowel disease; TECCU: Telemonitorización de la Enfermedad de Crohn y Colitis Ulcerosa or Telemonitoring of Crohn’s Disease and Ulcerative Colitis.

A total of 169 patients with complex IBD were enrolled in the study between September 15, 2020, and April 28, 2023. Of these, 11 patients were excluded: 4 due to screening failure, 2 due to a lack of baseline data, and 5 who withdrew before randomization. The remaining 158 patients proceeded to follow-up, with 74 (47%) in the G_TECCU group and 84 (53%) in the G_Control group. During the study period, 8 patients (3 from G_TECCU and 5 from G_Control) did not complete the 12-week follow-up. The reasons for this are detailed in Figure 2. As such, the 71 patients in the G_TECCU group and 79 patients in the G_Control group who completed the 12-week follow-up period were analyzed per protocol (N=150). Additionally, all randomized patients (n=158) were analyzed according to the intention-to-treat principle. The per-protocol population was used to summarize patient disposition and baseline characteristics (Figure 2; also see Multimedia Appendix 1 for the CONSORT-EHEALTH [Consolidated Standards of Reporting Trials of Electronic and Mobile Health Applications and Online Telehealth] checklist [35]).

The baseline demographic and clinical characteristics are presented in Table 1. The overall mean age of the participants was 37.8 (SD 18.7) years. Of the 150 patients, 83 (55%) were male and 67 (45%) were female. Among the included patients (n=150), 58 (39%) had UC, and 92 (61%) had CD, all of whom were capable of using the technology. The education levels of the patients were as follows: primary education (12/150, 8%), secondary education (70/150, 47%), and university education (68/150, 45%). Regarding employment status, 100 (66.7%) patients were actively employed, 19 (12.7%) were students, 17 (11.3%) were unemployed, 10 (6.7%) were retired, and 4 (2.7%) were houseworkers. At the onset of the study, 109 (72.7%) patients initiated treatment with biological agents (adalimumab in 55/109, 50.5%, patients), 25 (16.7%) with immunosuppressants (azathioprine in 23/25, 92%, patients), and 16 (10.7%) with combination therapy. No significant differences were observed in the baseline characteristics of patients according to the treatment initiated (immunosuppressants, biological agents, or combination therapy).

**Table 1:** Baseline characteristics of the study patients.

Characteristic		Total (N=150)	TECCU^a^ (n=71)	Standard care (n=79)
Age (years), mean (range)		37.76 (18-66)	38.83 (18-66)	36.80 (18-62)
**Gender, n (%)**				
	Female	67 (45)	34 (48)	33 (42)
	Male	83 (55)	37 (52)	46 (58)
**Education level, n (%)**				
	Primary	12 (8)	5 (7)	7 (9)
	Secondary	70 (47)	34 (48)	36 (46)
	University	68 (45)	32 (45)	36 (46)
**Smoking status, n (%)**				
	Current smoker	27 (18)	15 (21)	12 (15)
	Former smoker	50 (33)	27 (38)	23 (29)
	Nonsmoker	72 (48)	29 (41)	43 (54)
BMI (kg/m^2^), mean (SD)		23.65 (4.98)	23.35 (4.01)	23.93 (5.76)
**Disease profile, n (%)**				
	Ulcerative colitis	58 (39)	23 (32)	35 (44)
	Crohn disease	92 (61)	48 (68)	44 (56)
Prior IBD^b^-related surgery^c^, n (%)		3 (2)	1 (1)	2 (3)
**Previous treatments for IBD, n (%)**		129 (86)	61 (86)	68 (86)
	Immunosuppressors	66 (44)	30 (42)	36 (46)
	Biological agents	36 (24)	15 (21)	21 (27)
	Steroids	96 (64)	44 (62)	52 (66)
	Sulfasalazine and 5-aminosalicylates	68 (45)	30 (42)	38 (48)
	Other treatments	16 (11)	6 (8)	10 (13)
No previous treatment, n (%)		21 (14)	10 (14)	11 (14)
**Current treatment, n (%)**				
	Only immunosuppressors	25 (17)	8 (11)	17 (22)
	Only biological agents	109 (73)	58 (82)	51 (65)
	Combination therapy	16 (11)	5 (7)	11 (14)

^a^TECCU: Telemonitorización de la Enfermedad de Crohn y Colitis Ulcerosa or Telemonitoring of Crohn Disease and Ulcerative Colitis (represents a group of patients with inflammatory bowel disease receiving remote monitoring).

^b^IBD: inflammatory bowel disease.

^c^Within the past 2 months before study enrollment.

Regarding their clinical status (Table 2), 58 of 150 (38.7%) patients were in clinical remission at baseline; however, they were included in the study due to FC levels exceeding 200 µg/g or the presence of moderate to severe endoscopic activity. Concerning biological markers, the mean CRP value was 18.5 (SD 73.4) mg/L, while the mean FC level was 1631.1 (SD 2722.4) µg/g. Among the 24 patients with UC who entered the study following an endoscopic evaluation, 8 (33%) had a Mayo Index of 2, indicating moderate activity, while 16 patients (67%) had a Mayo Index of 3, indicating severe activity. Among the 34 patients with CD who underwent a baseline endoscopy, 27 (79%) exhibited ulcers. Furthermore, 61 of 150 (41%) patients demonstrated good adherence to medication (25/71, 35%, in the G_TECCU group and 36/79, 46%, in the G_Control group). Patient satisfaction, as measured by specific questionnaires, yielded a score of 87.3 (SD 11.2).

**Table 2:** Evaluation of disease activity, impact on patient’s quality of life and daily life, satisfaction with the medical care received, and medication adherence at baseline.

Characteristic		Total (N=150)	TECCU^a^ (n=71)	Standard care (n=79)
**Clinical activity** ^b^				
	SCCAI^c^<3 or HBI^d^<5 (but fecal calprotectin >200 μg/g or endoscopic activity), n (%)	58 (39)	33 (46)	25 (32)
	SCCAI^e^, mean (SD)	6.59 (3.05)	6.78 (2.37)	6.46 (3.45)
	Harvey-Bradshaw index^f^, mean (SD)	4.68 (3.11)	3.94 (2.97)	5.48 (3.10)
**Biological activity, mean (SD)**				
	C-reactive protein level (mg/L)	18.53 (73.43)	20.26 (90.96)	17.02 (54.13)
	Fecal calprotectin level (μg/g)	1631.1 (2722.4)	1418.7 (1977.1)	1614.9 (3322.9)
**Endoscopic activity, n/N (%)**				
	Mayo score of 2^g^	8/24 (33)	5/9 (56)	3/15 (20)
	Mayo score of 3^h^	16/24 (67)	4/9 (44)	12/15 (80)
	Patients with Crohn disease with ulcers	27/34 (79)	10/14 (71)	17/20 (85)
**Quality of life** ^i^				
	IBDQ-9^j^ score, mean (SD)	54.69 (20.46)	59.01 (21.79)	50.36 (19.76)
	Medication adherence^k^, n (%)	61 (41)	25 (35)	36 (46)
	Patient satisfaction score, mean (SD)	87.31 (11.17)	88.04 (10.44)	87.13 (11.84)
**Work productivity and activity impairment, median (IQR)**				
	Work hours missed	7.14 (0-100)	3.34 (0-66.7)	9.72 (0-100)
	Impairment while working	40.00 (0-75)	30.00 (0-70)	40.00 (10-80)
	Overall work impairment	50.00 (10-90)	41.21 (0-82)	50.00 (14.9-93)
	Activity impairment	50.00 (20-70)	40.00 (20-70)	50.00 (20-70)

^a^TECCU: *Telemonitorización de la Enfermedad de Crohn y Colitis Ulcerosa* or Telemonitoring of Crohn Disease and Ulcerative Colitis (represents a group of patients with inflammatory bowel disease receiving remote monitoring).

^b^Clinical remission was defined as HBI ≤4 for patients with Crohn disease or Walmsley score ≤2 for patients with ulcerative colitis.

^c^SCCAI: Simple Clinical Colitis Activity Index.

^d^HBI: Harvey-Bradshaw Index.

^e^Patients with ulcerative colitis: higher scores indicate worse clinical conditions.

^f^Patients with Crohn disease: score ranges from 0 to 19, where the higher the score the worse the clinical condition.

^g^Moderate disease.

^h^Severe disease.

^i^The higher the score, the better quality of life.

^j^IBDQ-9: 9-item Inflammatory Bowel Disease Questionnaire.

^k^Score ranges from 0 to 8, with scores of 8 reflectin*g* high adherence.

### Efficacy Assessment: Disease Activity

**Figure 3:**
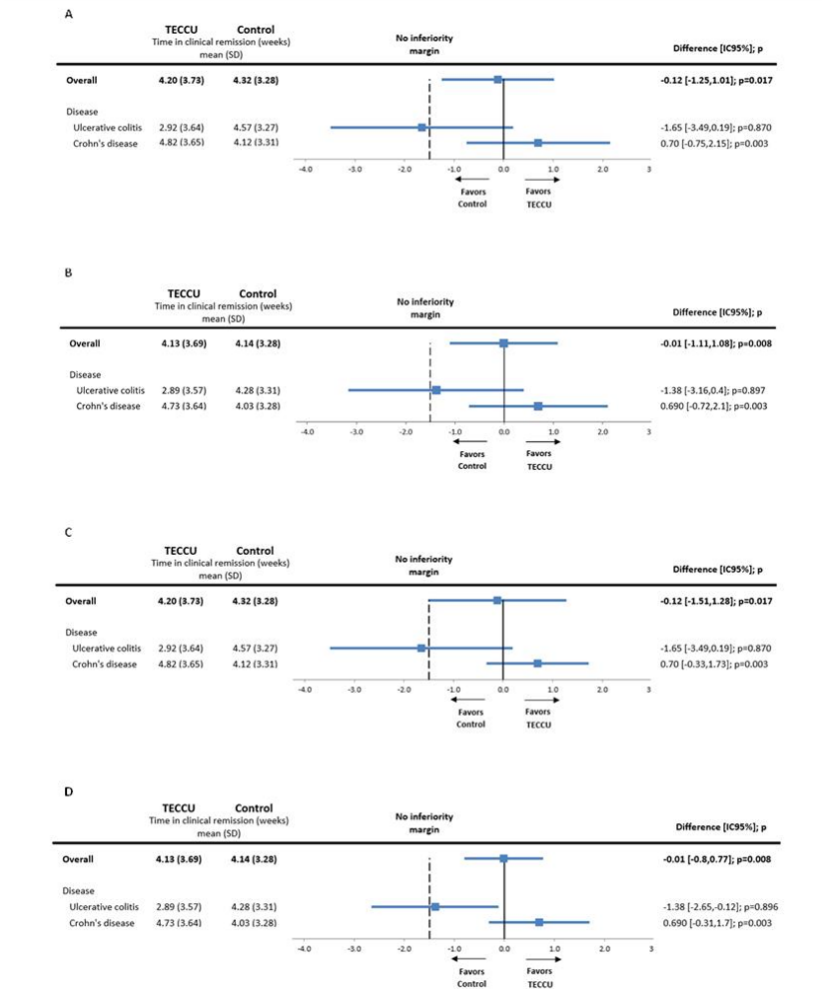
(A) Differences in the time in clinical remission between the 2 arms by the PP population. (B) Differences in the time in clinical remission between the 2 arms by the ITT population. (C) Differences in the time in clinical remission between the 2 arms by the PP population in multiplicity adjustments. (D) Differences in the time in clinical remission between the 2 arms by the ITT population in multiplicity adjustments. The data are shown for the whole cohort of patient analyzed and for specific subgroup (patients with UC or CD) analysis. Control: group of IBD patients receiving standard care with in-person visits; TECCU: group of IBD patients receiving remote monitoring. The dashed vertical line represents the non-inferiority margin of -1.5 weeks. CD: Crohn disease; ITT: intention to treat; PP: per-protocol; TECCU: Telemonitorización de la Enfermedad de Crohn y Colitis Ulcerosa or Telemonitoring of Crohn’s Disease and Ulcerative Colitis; UC: ulcerative colitis.

The mean time in clinical remission after the 12-week follow-up was 4.26 (SD 3.49) weeks for the entire study cohort. Based on the noninferiority statistics, the follow-up with TECCU was not inferior to standard care, with a mean time in remission of 4.20 (SD 3.73) weeks for the G_TECCU patients compared with 4.32 (SD 3.28) weeks for the G_Control patients (noninferiority *P*=.02; see Table 3). The mean difference in clinical remission between the 2 groups was –0.12 weeks, with a 95% CI of –1.25 to 1.01 (G_TECCU vs G_Control, *P*=.02; see Figure 3A).

**Table 3:** Time in clinical remission by the per-protocol population.

Time in clinical remission (weeks)		Participants, n	Mean (SD)	Median (IQR)	Range	Noninferiority test *P* value^a^	
**Overall**							.02^b^
	Total	150	4.26 (3.49)	4.42 (0.45-6.67)	0.00-14.29		
	TECCU^c^	71	4.20 (3.73)	4.33 (0.16-8.00)	0.00-14.29		
	Control^d^	79	4.32 (3.28)	4.61 (0.57-6.33)	0.00-12.43		
**Colitis ulcerative**							.87
	Total	58	3.92 (3.49)	4.00 (0.01-7.60)	0.00-10.86		
	TECCU	23	2.92 (3.64)	0.45 (0.00-4.78)	0.00-10.00		
	Control	35	4.57 (3.27)	4.71 (0.49-7.86)	0.00-10.86		
**Crohn disease**							.003^b^
	Total	92	4.48 (3.49)	4.57 (0.64-6.53)	0.00-14.29		
	TECCU	48	4.82 (3.65)	4.71 (0.69-8.08)	0.00-14.29		
	Control	44	4.12 (3.30)	4.41 (0.58-6.07)	0.00-12.43		

^a^Noninferiority limit=–1.5.

^b^Significant.

^c^TECCU: Telemonitorización de la Enfermedad de Crohn y Colitis Ulcerosa or Telemonitoring of Crohn Disease and Ulcerative Colitis (represents a group of patients with inflammatory bowel disease receiving remote monitoring).

^d^Control represents a group of patients with inflammatory bowel disease receiving standard care with in-person visits.

The subgroup analysis for patients with CD revealed a mean difference in clinical remission of +0.70 weeks for G_TECCU compared with G_Control (95% CI –0.75 to 2.15; *P*=.003). Given the statistical significance, the noninferiority of TECCU was confirmed in this subgroup. By contrast, the mean difference in clinical remission for patients with UC was –1.65 weeks between G_TECCU and G_Control (95% CI –3.49 to 0.19; *P*=.87), indicating that noninferiority of TECCU could not be confirmed in this cohort (Figure 3A). Similar results were observed in the intention-to-treat population and after adjusting for multiple tests (see Tables 4-6 and Figure 3B-3D).

After 12 weeks, the mean SCCAI significantly improved in both the G_TECCU (mean change –3.78, SD 3.16, *P*<.001) and G_Control patients with UC (mean change –4.11, SD 4.31; *P*<.001). Similarly, the mean HBI value also showed a significant improvement in G_TECCU patients (mean change –1.42, SD 2.69; *P*<.001) and G_Control patients (mean change –2.07, SD 4.06; *P*=.002). However, the improvements in SCCAI (*P*=.27) and HBI (*P*=.26) scores were not significantly different between the 2 intervention arms.

**Figure 4:**
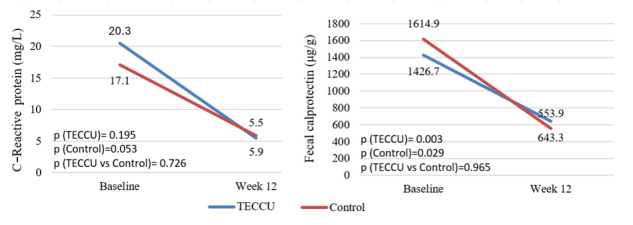
Evolution of the mean of (A) C-reactive protein and (B) fecal calprotectin levels over the study period in the 2 arms. Control: group receiving standard care with in-person visits; TECCU: group receiving remote monitoring. TECCU: Telemonitorización de la Enfermedad de Crohn y Colitis Ulcerosa or Telemonitoring of Crohn’s Disease and Ulcerative Colitis.

Disease activity was also assessed based on CRP and FC levels (Figure 4). The mean CRP value significantly decreased after 12 weeks compared with baseline in both groups, with a mean reduction of –15.40 (SD 90.15) mg/L (*P*=.19) in the G_TECCU group and –13.16 (SD 54.61) mg/L (*P*=.05) in the G_Control group. However, this improvement in CRP did not significantly differ between the 2 groups (G_TECCU vs G_Control: *P*=.73). Similarly, the mean FC levels were significantly lower after 12 weeks compared with baseline in both study groups. The G_TECCU group experienced a mean reduction of 832.3 (SD 1825.0) mg/L (*P*=.003), while the G_Control group had a mean reduction of 1073.5 (SD 3105.7) mg/L (*P*=.03). However, there were no significant differences between the 2 groups (*P*=.96).

**Table 4:** Time in clinical remission by the intention-to-treat population.

Time in clinical remission (weeks)			Participants, n		Mean (SD)		Median (IQR)		Range		Noninferiority test *P* value^a^	
**Overall**												.008^b^
	Total	158		4.14 (3.47)		4.33 (0.31-6.33)		0.00-14.29				
	TECCU^c^	74		4.13 (3.69)		4.23 (0.16-7.70)		0.00-14.29				
	Control^d^	84		4.14 (3.28)		4.41 (0.42-6.07)		0.00-12.43				
**Colitis ulcerative**												.90
	Total	62		3.74 (3.45)		4.00 (0.00-5.86)		0.00-10.86				
	TECCU	24		2.89 (3.57)		0.60 (0.00-4.60)		0.00-10.00				
	Control	38		4.28 (3.31)		4.65 (0.29-7.60)		0.00-10.86				
**Crohn disease**												.003^b^
	Total	96		4.39 (3.47)		4.53 (0.61-6.36)		0.00-14.29				
	TECCU	50		4.73 (3.64)		4.71 (0.67-8.00)		0.00-14.29				
	Control	46		4.03 (3.28)		4.33 (0.57-6.01)		0.00-12.43				

^a^Noninferiority limit=–1.5.

^b^Significant.

^c^TECCU: *Telemonitorización de la Enfermedad de Crohn y Colitis Ulcerosa* or Telemonitoring of Crohn Disease and Ulcerative Colitis (represents a group of patients with inflammatory bowel disease receiving remote monitoring).

^d^Control represents a group of patients with inflammatory bowel disease receiving standard care with in-person visits.

**Table 5:** Time in clinical remission by the per-protocol population in multiplicity adjustments.

Time in clinical remission (weeks)		Participants, n	Mean (SD)	Median (IQR)	Range	Noninferiority test *P* value^a^	
**Overall**							.02^b^
	Total	150	4.26 (3.49)	4.42 (0.45-6.67)	0.00-14.29		
	TECCU^c^	71	4.20 (3.73)	4.33 (0.16-8.00)	0.00-14.29		
	Control^d^	79	4.32 (3.28)	4.61 (0.57-6.33)	0.00-12.43		
**Colitis ulcerative**							.87
	Total	58	3.92 (3.49)	4.00 (0.01-7.60)	0.00-10.86		
	TECCU	23	2.92 (3.64)	0.45 (0.00-4.78)	0.00-10.00		
	Control	35	4.57 (3.27)	4.71 (0.49-7.86)	0.00-10.86		
**Crohn disease**							.003^b^
	Total	92	4.48 (3.49)	4.57 (0.64-6.53)	0.00-14.29		
	TECCU	48	4.82 (3.65)	4.71 (0.69-8.08)	0.00-14.29		
	Control	44	4.12 (3.30)	4.41 (0.58-6.07)	0.00-12.43		

^a^Noninferiority limit=–1.5.

^b^Significant.

^c^TECCU: *Telemonitorización de la Enfermedad de Crohn y Colitis Ulcerosa* or Telemonitoring of Crohn Disease and Ulcerative Colitis (represents a group of patients with inflammatory bowel disease receiving remote monitoring).

^d^Control represents a group of patients with inflammatory bowel disease receiving standard care with in-person visits.

**Table 6:** Time in clinical remission by the intention-to-treat population by multiplicity adjustments.

Time in clinical remission (weeks)			Participants, n		Mean (SD)		Median (IQR)		Range		Noninferiority test *P* value^a^	
**Overall**												.008^b^
	Total	158		4.14 (3.47)		4.33 (0.31-6.33)		0.00-14.29				
	TECCU^c^	74		4.13 (3.69)		4.23 (0.16-7.70)		0.00-14.29				
	Control^d^	84		4.14 (3.28)		4.41 (0.42-6.07)		0.00-12.43				
**Colitis ulcerative**												.90
	Total	62		3.74 (3.45)		4.00 (0.00-5.86)		0.00-10.86				
	TECCU	24		2.89 (3.57)		0.60 (0.00-4.60)		0.00-10.00				
	Control	38		4.28 (3.31)		4.65 (0.29-7.60)		0.00-10.86				
**Crohn disease**												.003^b^
	Total	96		4.39 (3.47)		4.53 (0.61-6.36)		0.00-14.29				
	TECCU	50		4.73 (3.64)		4.71 (0.67-8.00)		0.00-14.29				
	Control	46		4.03 (3.28)		4.33 (0.57-6.01)		0.00-12.43				

^a^Noninferiority limit=–1.5.

^b^Significant.

^c^TECCU: *Telemonitorización de la Enfermedad de Crohn y Colitis Ulcerosa* or Telemonitoring of Crohn Disease and Ulcerative Colitis (represents a group of patients with inflammatory bowel disease receiving remote monitoring).

^d^Control represents a group of patients with inflammatory bowel disease receiving standard care with in-person visits.

The difference in the mean change in CRP levels between the groups after 12 weeks was –2.25 mg/L for G_TECCU compared with G_Control (95% CI –28.19 to 23.69); however, noninferiority of TECCU could not be demonstrated (noninferiority *P*=.73). Similarly, noninferiority was not demonstrated for the reduction in FC levels among G_TECCU patients, with a mean change difference of 241.2 μg/g compared with G_Control (95% CI –806.9 to 1289.2, noninferiority *P*=.96).

### Health-Related Quality of Life

**Figure 5:**
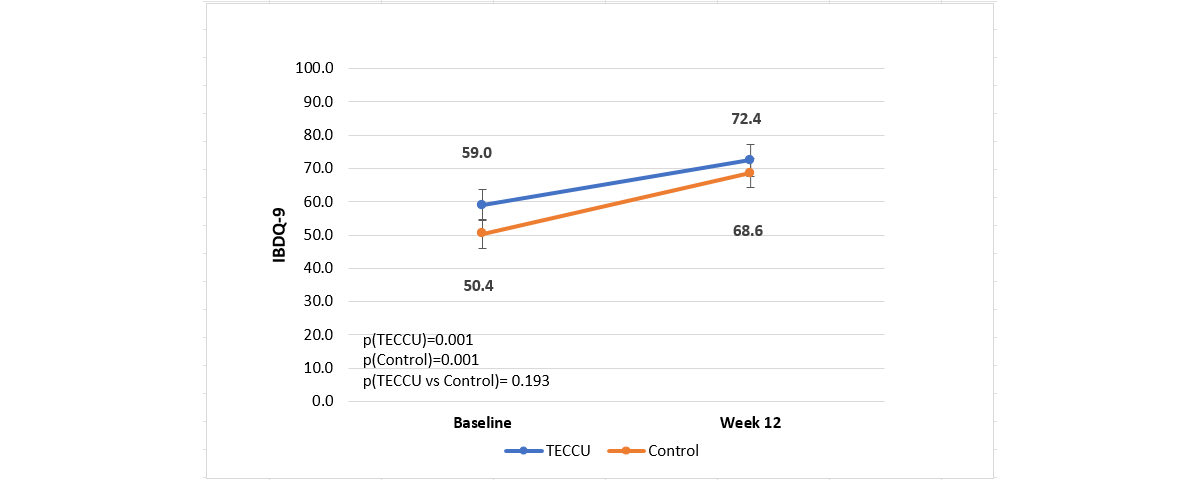
Evolution of the IBDQ-9 score over the study period in the 2 arms. Control: group receiving standard care with in-person visits; TECCU: group receiving remote monitoring. IBDQ-9: 9-item Inflammatory Bowel Disease Questionnaire; TECCU: Telemonitorización de la Enfermedad de Crohn y Colitis Ulcerosa or Telemonitoring of Crohn’s Disease and Ulcerative Colitis.

The mean IBDQ-9 scores increased from 59.01 (SD 21.8) to 72.44 (SD 20.3) in G_TECCU patients, indicating a mean improvement of 13.44 points (SD 19.1, *P*<.001). In G_Control patients, scores improved from 50.36 (SD 19.8) to 68.58 (SD 20.2), reflecting a mean improvement of 18.23 points (SD 22.9; *P*=.001; Figure 5). However, because the mean difference between the 2 groups was –4.79 (95% CI –12.9 to 3.3; *P*=.90) and the CI crossed the noninferiority margin, the noninferiority of TECCU in improving the IBDQ-9 score could not be demonstrated.

### Work Productivity and Activity Impairment

Regarding the impact of the disease on work productivity and daily activities, a significant improvement in WPAI scores was observed at 12 weeks compared with baseline in the G_Control arm (*P=*.001 for all scores). By contrast, TECCU only demonstrated a significant improvement in impairment while working (mean improvement –17.74, SD 39.65; *P*=.003) and in activity impairment (mean improvement –14.49, SD 30.69; *P*=.002). However, no significant differences between the 2 arms were found in work hours missed due to health problems (*P=*.21), impairment while working (*P=*.38), overall work impairment (*P=*.19), or activity impairment (*P=*.11).

### Medication Adherence

**Figure 6:**
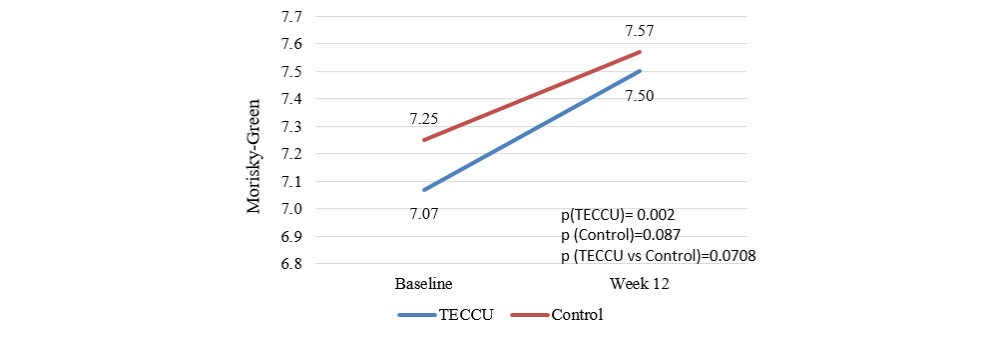
Evolution of the MMAS-8 score over the study period in the 2 arms. Control, group receiving standard care with in-person visits; TECCU, group receiving remote monitoring. MMAS-8: 8-item Morisky Medication Adherence Scale; TECCU: Telemonitorización de la Enfermedad de Crohn y Colitis Ulcerosa or Telemonitoring of Crohn’s Disease and Ulcerative Colitis.

After 12 weeks, the proportion of patients adhering to their medication increased significantly in the G_TECCU group (rising from 25/71, 35%, to 48/71, 68%; *P*=.001) and in the G_Control group (increasing from 36/79, 46%, to 58/79, 73%; *P*=.001). However, this improvement in medication adherence did not differ significantly between the 2 arms (G_TECCU vs G_Control: *P*=.46). Additionally, the MMAS-8 score in G_TECCU patients improved significantly after 12 weeks, with a mean baseline value increasing from 7.07 (SD 1.03) to 7.50 (SD 0.86), representing a mean improvement of 0.48 (SD 1.6, *P*=.002). Similarly, the MMAS-8 score improved significantly in the G_Control group after 12 weeks, rising from a mean baseline value of 7.25 (SD 1.1) to 7.57 (SD 1.2), reflecting a mean improvement of 0.29 (SD 1.3, *P*=.09; Figure 6). The difference in the mean change of the MMAS-8 score between the 2 arms was 0.19 (95% CI –0.24 to 0.63; *P*=.39), indicating a trend toward statistical significance regarding the noninferiority of G_TECCU compared with G_Control (*P*=.07).

Changes in medication were made for 24 of the 150 patients (16%) during the study period, with no significant differences observed in the number of patients who changed their medication between the groups (G_TECCU: 12/71, 17%, patients vs G_Control: 12/79, 15%, patients; *P*=.77).

### Patient Satisfaction

**Figure 7:**
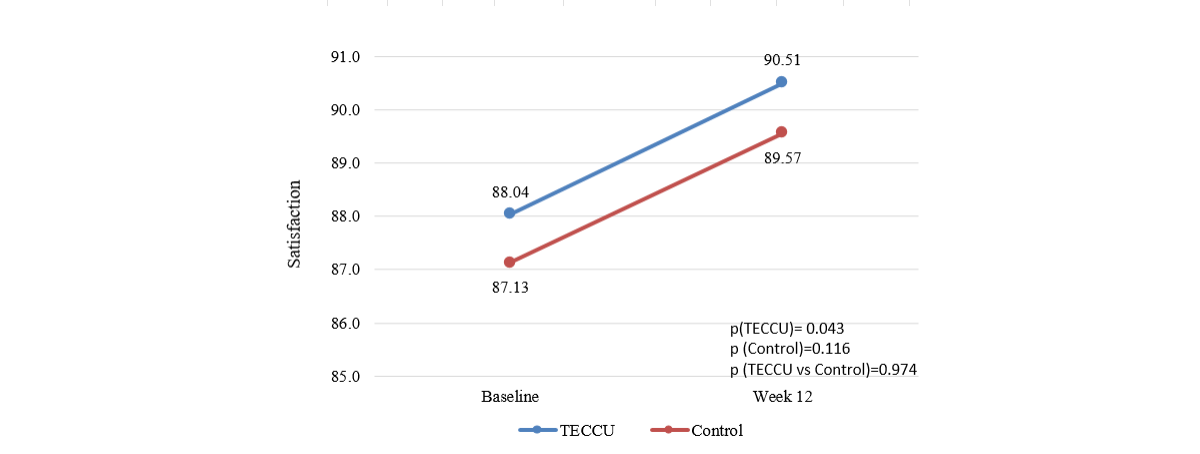
Evolution of the patient satisfaction score over the study period in the 2 arms. Control: group receiving standard care with in-person visits; TECCU: group receiving remote monitoring. CSQ: Client Satisfaction Questionnaire; TECCU: Telemonitorización de la Enfermedad de Crohn y Colitis Ulcerosa or Telemonitoring of Crohn’s Disease and Ulcerative Colitis.

In the G_TECCU group, the mean satisfaction questionnaire score improved from 88.04 (SD 10.3) to 90.51 (SD 9.7; *P=*.04), while in the G_Control group, it increased from 87.13 (SD 12.3) to 89.57 (SD 8.5; *P=*.12). The mean improvement in satisfaction scores was 2.47 (SD 8.6) for G_TECCU (*P*=.04) and 2.43 (SD 11.2) for G_Control (*P*=.12); however, the noninferiority test between the groups was not significant (*P*=.97; Figure 7).

### Use of Health Care Resources and Safety

Emergency department visits were significantly higher in the G_TECCU group compared with the G_Control group (13/71, 18%, G_TECCU vs 5/79, 6%, G_Control; *P*=.02). However, the intervention did not result in significant differences in unscheduled outpatient visits (19/71, 27%, G_TECCU vs 16/79, 20%, G_Control; *P*=.35) or hospitalizations (5/71, 7%, G_TECCU vs 2/79, 3%, G_Control; *P*=.19). A total of 70 AEs were recorded in the G_TECCU group in 39 of 71 (55%) patients, while 60 AEs were noted in the G_Control group in 31 of 79 (39%) patients. However, the differences between groups did not reach statistical significance (*P*=.054).

Among these AEs, 5 resulted in patient loss during the follow-up period, with 2 in the G_TECCU group and 3 in the G_Control group. A total of 17 AEs were related to medication, comprising 8 of 71 (11%) patients in the G_TECCU group and 9 of 79 (11%) patients in the G_Control group, with no significant differences between the groups (*P*=.98). Notably, none of the patients died during this period.

## Discussion

### Principal Findings and Comparison With Prior Work

To the best of our knowledge, this is the first trial using a noninferiority design to compare telemonitoring with standard care for patients with moderate-to-severe IBD. In previous studies, telemonitoring was shown to be safe and to improve both health outcomes and costs [8,9,11,13-15]. In fact, telemonitoring can meet the treat-to-target strategy by addressing many variables of the STRIDE-II recommendations [18], but it failed to demonstrate superiority over standard care in many studies [8,10,11,13,14,17]. Nonetheless, noninferiority would also be an acceptable goal if, in return, telemonitoring improves the efficiency of care. In light of the positive results obtained with TECCU in a pilot trial carried out on patients with active IBD [20], here we demonstrate on a nationwide scale that telemonitoring with the TECCU app is not inferior to standard care in inducing and maintaining remission over 12 weeks.

The main outcome of this study was the time patients remained in remission, which is relevant because we evaluated the evolution of clinical activity throughout the follow-up period, not just the clinical status at the end of the trial, as reported thus far [13,15,19,20,36]. The mean period in remission with telemonitoring was 4.20 weeks, a relatively short time that was probably related to the selection of patients during the induction phase of treatment. Then, in this work, we evaluated the first 12 weeks after initiating biological agents or immunosuppressants to manage persistent disease activity or a flare-up. Indeed, the mean time in remission was essentially the same as that achieved through standard care, and its distribution in each group was sufficiently similar to demonstrate noninferiority.

A subgroup analysis confirmed the noninferiority of TECCU for patients with CD, but it was inconclusive for patients with UC. Furthermore, the improvement in clinical activity was consistent with the reduction in the markers of inflammation (CRP and FC) over time. These data imply that patients with active IBD benefit from both telemonitoring and standard care, and while telemonitoring may not improve disease activity compared with in-person visits, it may enhance other health outcomes, reduce costs, decrease time off work, and lower the number of hospital visits, among other benefits.

In this sense, the TECCU app previously demonstrated greater cost-effectiveness than standard care in a similar subset of patients [24,25], as subsequently reproduced in other settings [37,38]. Therefore, telemonitoring stands out as an interesting alternative to standard care in the follow-up of patients with IBD, whether they are experiencing active disease or not. Either of these options could be used complementarily during a patient’s follow-up, based on the preferences of both patients and health care providers in each case. In this regard, telemonitoring apps can leverage the advancements achieved during the COVID-19 pandemic [39].

As witnessed here, enhanced QoL and medication adherence are generally inversely correlated with disease activity [7]. The IBDQ-9 and the MMAS-8 medication adherence scores improved in both the TECCU and control groups after 12 weeks, indicating a trend toward statistical significance in the noninferiority of G_TECCU compared with G_Control in medication adherence. The improvement in HRQoL could be attributed to the reduction in disease activity in both groups following the escalation of medication, rather than solely to the follow-up method itself. Furthermore, the enhancement in HRQoL was similarly linked to a significant improvement in work productivity and a reduction in activity impairment (as measured by the WPAI) at 12 weeks compared with baseline in both groups.

Another notable finding was that a higher proportion of patients in the G_TECCU group had emergency department visits. However, there were no significant differences between the groups concerning unscheduled outpatient visits, hospitalizations, or AEs. Taken together, these results indicate that the telemonitoring follow-up schedule should be tailored to each patient, and the learning curve for implementing this approach may vary between centers. This aspect will be further analyzed after the completion of the 12-month follow-up period of this trial.

The efficacy of telemonitoring lies in its capacity to deliver interventions tailored to the varying clinical scenarios presented by patients who are open to using remote care, rather than solely focusing on how these interventions are provided. In fact, various patient profiles have been identified as predictors of failure in a telemedicine context, partly due to the diverse demographic and socioeconomic characteristics of the patients [40]. In this context, patient satisfaction remained stable in both the G_TECCU and G_Control groups in our trial, with values around 90% after 12 weeks. It is important to note that the G_TECCU group had received standard care before being enrolled in the study, and the switch to telemonitoring did not diminish their level of satisfaction, even though their disease activity was uncontrolled at that time.

### Strengths and Limitations

The main strength of this study is that it was designed as a randomized controlled trial, allowing us to assess the impact of a web-based telemonitoring system on disease outcomes in the follow-up of IBD and to compare this with standard care. Additionally, we selected patients during the induction phase of biological agents or those initiating immunosuppressants due to active IBD or a flare-up, a specific population of IBD that has been underrepresented in previous studies [8,13,15,19,41].

Moreover, the use of validated indices of activity and biological markers (CRP and FC) that correlate well with endoscopic activity [42-45] added value to the assessment of remote monitoring. Additionally, the follow-up schedule was consistent across both arms and was adapted to daily practices in the management of IBD, which enhances the reproducibility of the results obtained. Finally, this nationwide study includes patients recruited from 24 hospitals across Spain, further enhancing the external validity of our findings.

Among the limitations of this study, the follow-up period was only 12 weeks. While this time frame may be adequate to evaluate the induction phase of therapies, it may be too short to fully assess the maintenance period, especially because patients and physicians need some time to learn how to use the telemonitoring platform. Therefore, trials with longer follow-up periods will be necessary to confirm the efficacy of this web-based system in improving long-term disease outcomes. Indeed, the follow-up of the patients enrolled in this study continues, and there are intentions to extend this period to 52 weeks for as many of these patients as possible.

Besides, the sample size may be too small to compare disease outcomes in specific patient subgroups (eg, patients with UC or CD and those receiving different medications). In addition, given the nature of the interventions assessed, neither the patients nor the researchers were blinded to the intervention; however, the data obtained were analyzed by an independent statistician who was blind to group identification. Finally, we excluded patients with active perianal disease, ileo-rectal/ileo-pouch anal anastomosis, or stoma; therefore, the impact of TECCU on surgical patients, in whom physical examination is very important, remains unclear.

### Conclusions

This Spanish multicenter trial conducted on behalf of GETECCU studied patients with active IBD who began using biological agents or immunosuppressant drugs, demonstrating that the TECCU app is not inferior to standard care for achieving and maintaining remission in the short term. Noninferiority could represent a meaningful advancement if, as seen in other studies, telemonitoring improves additional health outcomes and reduces costs. The TECCU app is also associated with improved medication adherence and HRQoL; however, a reduction in costs still needs to be demonstrated over a longer follow-up period.

## Appendix

Multimedia Appendix 1 CONSORT E-HEALTH checklist.

## Abbreviations

AE: adverse event
CD: Crohn disease
CONSORT-EHEALTH: Consolidated Standards of Reporting Trials of Electronic and Mobile Health Applications and Online Telehealth
CRP: C-reactive protein
FC: fecal calprotectin
HBI: Harvey-Bradshaw Index
HRQoL: health-related quality of life
IBD: inflammatory bowel disease
IBDQ-9: 9-item Inflammatory Bowel Disease Questionnaire
mHealth: mobile health
MMAS-8: 8-item Morisky Medication Adherence Scale
SCCAI: Simple Clinical Colitis Activity Index
TECCU: Telemonitorización de la Enfermedad de Crohn y Colitis Ulcerosa or Telemonitoring of Crohn Disease and Ulcerative Colitis
UC: ulcerative colitis
WPAI: Work productivity and activity impairment

## Data Availability

The data presented in this article will be shared on reasonable request to the corresponding author.

## References

[1] Høivik Marte Lie, Moum B, Solberg IC, Henriksen M, Cvancarova M, Bernklev T, IBSEN Group (2013). Work disability in inflammatory bowel disease patients 10 years after disease onset: results from the IBSEN Study. Gut.

[2] Kappelman MD, Porter CQ, Galanko JA, Rifas-Shiman SL, Ollendorf DA, Sandler RS, Finkelstein JA (2011). Utilization of healthcare resources by U.S. children and adults with inflammatory bowel disease. Inflamm Bowel Dis.

[3] Ng SC, Shi HY, Hamidi N, Underwood FE, Tang W, Benchimol EI, Panaccione R, Ghosh S, Wu JCY, Chan FKL, Sung JJY, Kaplan GG (2017). Worldwide incidence and prevalence of inflammatory bowel disease in the 21st century: a systematic review of population-based studies. Lancet.

[4] Molodecky NA, Soon IS, Rabi DM, Ghali WA, Ferris M, Chernoff G, Benchimol EI, Panaccione R, Ghosh S, Barkema HW, Kaplan GG (2012). Increasing incidence and prevalence of the inflammatory bowel diseases with time, based on systematic review. Gastroenterology.

[5] Chaparro M, Garre A, Núñez Ortiz Andrea, Diz-Lois Palomares María Teresa, Rodríguez Cristina, Riestra S, Vela Milagros, Benítez José Manuel, Fernández Salgado Estela, Sánchez Rodríguez Eugenia, Hernández Vicent, Ferreiro-Iglesias Rocío, Ponferrada Díaz Ángel, Barrio Jesús, Huguet José María, Sicilia Beatriz, Martín-Arranz María Dolores, Calvet Xavier, Ginard Daniel, Alonso-Abreu Inmaculada, Fernández-Salazar Luis, Varela Trastoy Pilar, Rivero Montserrat, Vera-Mendoza Isabel, Vega Pablo, Navarro Pablo, Sierra Mónica, Cabriada José Luis, Aguas Mariam, Vicente Raquel, Navarro-Llavat Mercè, Echarri Ana, Gomollón Fernando, Guerra Del Río Elena, Piñero Concepción, Casanova María José, Spicakova Katerina, Ortiz de Zarate Jone, Torrella Cortés Emilio, Gutiérrez Ana, Alonso-Galán Horacio, Hernández-Martínez Álvaro, Marrero José Miguel, Lorente Poyatos Rufo, Calafat Margalida, Martí Romero Lidia, Robledo Pilar, Bosch Orencio, Jiménez Nuria, Esteve Comas María, Duque José María, Fuentes Coronel Ana María, Josefa Sampedro Manuela, Sesé Abizanda Eva, Herreros Martínez Belén, Pozzati Liliana, Fernández Rosáenz Hipólito, Crespo Suarez Belén, López Serrano Pilar, Lucendo Alfredo J, Muñoz Vicente Margarita, Bermejo Fernando, Ramírez Palanca José Joaquín, Menacho Margarita, Carmona Amalia, Camargo Raquel, Torra Alsina Sandra, Maroto Nuria, Nerín de la Puerta Juan, Castro Elena, Marín-Jiménez Ignacio, Botella Belén, Sapiña Amparo, Cruz Noelia, Forcelledo José Luis F, Bouhmidi Abdel, Castaño-Milla Carlos, Opio Verónica, Nicolás Isabel, Kutz Marcos, Abraldes Bechiarelli Alfredo, Gordillo Jordi, Ber Yolanda, Torres Domínguez Yolanda, Novella Durán María Teresa, Rodríguez Mondéjar Silvia, Martínez-Cerezo Francisco J, Kolle Lilyan, Sabat Miriam, Ledezma Cesar, Iyo Eduardo, Roncero Óscar, Irisarri Rebeca, Lluis Laia, Blázquez Gómez Isabel, Zapata Eva María, José Alcalá María, Martínez Pascual Cristina, Montealegre María, Mata Laura, Monrobel Ana, Hernández Camba Alejandro, Hernández Luis, Tejada María, Mir Alberto, Galve María Luisa, Soler Marta, Hervías Daniel, Gómez-Valero José Antonio, Barreiro-de Acosta Manuel, Rodríguez-Artalejo Fernando, García-Esquinas Esther, Gisbert Javier P, The EpidemIBD Study Group Of Geteccu (2021). Incidence, clinical characteristics and management of inflammatory bowel disease in Spain: large-scale epidemiological study. J Clin Med.

[6] Aguas Peris M, Del Hoyo J, Bebia P, Faubel R, Barrios A, Bastida G, Valdivieso B, Nos P (2015). Telemedicine in inflammatory bowel disease: opportunities and approaches. Inflamm Bowel Dis.

[7] Del Hoyo J, Millán Mónica, Garrido-Marín Alejandro, Aguas M (2023). Are we ready for telemonitoring inflammatory bowel disease? A review of advances, enablers, and barriers. World J Gastroenterol.

[8] Elkjaer M, Shuhaibar M, Burisch J, Bailey Y, Scherfig H, Laugesen B, Avnstrøm Søren, Langholz E, O'Morain C, Lynge E, Munkholm P (2010). E-health empowers patients with ulcerative colitis: a randomised controlled trial of the web-guided 'Constant-care' approach. Gut.

[9] Carlsen K, Jakobsen C, Houen G, Kallemose T, Paerregaard A, Riis LB, Munkholm P, Wewer V (2017). Self-managed eHealth disease monitoring in children and adolescents with inflammatory bowel disease: a randomized controlled trial. Inflamm Bowel Dis.

[10] Carlsen K, Houen G, Jakobsen C, Kallemose T, Paerregaard A, Riis LB, Munkholm P, Wewer V (2017). Individualized infliximab treatment guided by patient-managed eHealth in children and adolescents with inflammatory bowel disease. Inflamm Bowel Dis.

[11] Heida A, Dijkstra A, Muller Kobold Anneke, Rossen JW, Kindermann A, Kokke F, de Meij Tim, Norbruis O, Weersma RK, Wessels M, Hummel T, Escher J, van Wering Herbert, Hendriks D, Mearin L, Groen H, Verkade HJ, van Rheenen Patrick F (2018). Efficacy of home telemonitoring versus conventional follow-up: a randomized controlled trial among teenagers with inflammatory bowel disease. J Crohns Colitis.

[12] Yin AL, Hachuel D, Pollak JP, Scherl EJ, Estrin D (2019). Digital health apps in the clinical care of inflammatory bowel disease: scoping review. J Med Internet Res.

[13] de Jong Marin J, van der Meulen-de Jong Andrea E, Romberg-Camps MJ, Becx MC, Maljaars JP, Cilissen M, van Bodegraven Ad A, Mahmmod N, Markus T, Hameeteman WM, Dijkstra G, Masclee AA, Boonen A, Winkens B, van Tubergen Astrid, Jonkers DM, Pierik MJ (2017). Telemedicine for management of inflammatory bowel disease (myIBDcoach): a pragmatic, multicentre, randomised controlled trial. Lancet.

[14] Johnson MW, Lithgo K, Price T (2013). OC-080 Ibd-Sshamp (Supported, Self help and Management Programme); UK’s first internet based remote management system for managing stable IBD. Gut.

[15] Cross RK, Langenberg P, Regueiro M, Schwartz DA, Tracy JK, Collins JF, Katz J, Ghazi L, Patil SA, Quezada SM, Beaulieu D, Horst SN, Russman K, Riaz M, Jambaulikar G, Sivasailam B, Quinn CC (2019). A randomized controlled trial of TELEmedicine for patients with inflammatory bowel disease (TELE-IBD). Am J Gastroenterol.

[16] Zhen J, Marshall JK, Nguyen GC, Atreja A, Narula N (2021). Impact of digital health monitoring in the management of inflammatory bowel disease. J Med Syst.

[17] van Deen Welmoed K, Spiro A, Burak Ozbay A, Skup M, Centeno A, Duran N, Lacey Precious N, Jatulis Darius, Esrailian Eric, van Oijen Martijn G H, Hommes Daniel W (2017). The impact of value-based healthcare for inflammatory bowel diseases on healthcare utilization: a pilot study. Eur J Gastroenterol Hepatol.

[18] Turner D, Ricciuto A, Lewis A, D'Amico F, Dhaliwal J, Griffiths AM, Bettenworth D, Sandborn WJ, Sands BE, Reinisch W, Schölmerich Jürgen, Bemelman W, Danese S, Mary JY, Rubin D, Colombel J, Peyrin-Biroulet L, Dotan I, Abreu MT, Dignass A, International Organization for the Study of IBD (2021). STRIDE-II: An Update on the Selecting Therapeutic Targets in Inflammatory Bowel Disease (STRIDE) Initiative of the International Organization for the Study of IBD (IOIBD): determining therapeutic goals for treat-to-target strategies in IBD. Gastroenterology.

[19] Cross RK, Cheevers N, Rustgi A, Langenberg P, Finkelstein J (2012). Randomized, controlled trial of home telemanagement in patients with ulcerative colitis (UC HAT). Inflamm Bowel Dis.

[20] Del Hoyo J, Nos P, Faubel R, Muñoz Diana, Domínguez David, Bastida G, Valdivieso B, Correcher M, Aguas M (2018). A web-based telemanagement system for improving disease activity and quality of life in patients with complex inflammatory bowel disease: pilot randomized controlled trial. J Med Internet Res.

[21] Piaggio G, Elbourne DR, Pocock SJ, Evans SJW, Altman DG, CONSORT Group (2012). Reporting of noninferiority and equivalence randomized trials: extension of the CONSORT 2010 statement. JAMA.

[22] Jaikumar V Non-inferiority trials: understanding the concepts - tutorials and fundamentals. Stud 4 Best Evid.

[23] Aguas M, Del Hoyo J, Faubel R, Muñoz Diana, Domínguez David, Bastida G, Navarro B, Barrios A, Valdivieso B, Correcher M, Nos P (2018). A web-based telemanagement system for patients with complex inflammatory bowel disease: protocol for a randomized controlled clinical trial. JMIR Res Protoc.

[24] Del Hoyo J, Nos P, Bastida G, Faubel R, Muñoz Diana, Garrido-Marín Alejandro, Valero-Pérez Elena, Bejar-Serrano S, Aguas M (2019). Telemonitoring of Crohn's disease and ulcerative colitis (TECCU): cost-effectiveness analysis. J Med Internet Res.

[25] Del Hoyo J, Aguas M (2021). Cost-effectiveness of telemedicine-directed specialized vs standard care for patients with inflammatory bowel diseases in a randomized trial. Clin Gastroenterol Hepatol.

[26] Magro F, Gionchetti P, Eliakim R, Ardizzone S, Armuzzi A, Barreiro-de Acosta M, Burisch J, Gecse KB, Hart AL, Hindryckx P, Langner C, Limdi JK, Pellino G, Zagórowicz Edyta, Raine T, Harbord M, Rieder F, European Crohn’sColitis Organisation [ECCO] (2017). Third European Evidence-based Consensus on Diagnosis and Management of Ulcerative Colitis. Part 1: definitions, diagnosis, extra-intestinal manifestations, pregnancy, cancer surveillance, surgery, and ileo-anal pouch disorders. J Crohns Colitis.

[27] Gomollón Fernando, Dignass A, Annese V, Tilg H, Van Assche G, Lindsay JO, Peyrin-Biroulet L, Cullen GJ, Daperno M, Kucharzik T, Rieder F, Almer S, Armuzzi A, Harbord M, Langhorst J, Sans M, Chowers Y, Fiorino G, Juillerat P, Mantzaris GJ, Rizzello F, Vavricka S, Gionchetti P (2017). 3rd European Evidence-based Consensus on the Diagnosis and Management of Crohn's Disease 2016: part 1: diagnosis and medical management. J Crohns Colitis.

[28] Del Hoyo J, Nos P, Faubel R, Bastida G, Muñoz Diana, Valero-Pérez Elena, Garrido-Marín Alejandro, Bella P, Peña Beatriz, Savini C, Aguas M (2020). Adaptation of TECCU app based on patients´ perceptions for the telemonitoring of inflammatory bowel disease: a qualitative study using focus groups. Int J Environ Res Public Health.

[29] Sicilia B, García-López Santiago, González-Lama Yago, Zabana Y, Hinojosa J, Gomollón Fernando, Grupo Español de T rabajo de Enfermedad de Crohn (2020). GETECCU 2020 guidelines for the treatment of ulcerative colitis. Developed using the GRADE approach. Gastroenterol Hepatol.

[30] Harvey RF, Bradshaw JM (1980). A simple index of Crohn's-disease activity. Lancet.

[31] Walmsley RS, Ayres RC, Pounder RE, Allan RN (1998). A simple clinical colitis activity index. Gut.

[32] Vergara M, Montserrat A, Casellas F, Villoria A, Suarez D, Maudsley M, Gallardo O, Ricart E, Calvet X (2011). A new validation of the Spanish Work Productivity and Activity Impairment Questionnaire-Crohn's disease version. Value Health.

[33] Berlowitz DR, Foy CG, Kazis LE, Bolin LP, Conroy MB, Fitzpatrick P, Gure TR, Kimmel PL, Kirchner K, Morisky DE, Newman J, Olney C, Oparil S, Pajewski NM, Powell J, Ramsey T, Simmons DL, Snyder J, Supiano MA, Weiner DE, Whittle J, SPRINT Research Group (2017). Effect of intensive blood-pressure treatment on patient-reported outcomes. N Engl J Med.

[34] Bress Adam P, Bellows Brandon K, King JB, Hess Rachel, Beddhu Srinivasan, Zhang Zugui, Berlowitz Dan R, Conroy Molly B, Fine Larry, Oparil Suzanne, Morisky Donald E, Kazis Lewis E, Ruiz-Negrón Natalia, Powell Jamie, Tamariz Leonardo, Whittle Jeff, Wright Jackson T, Supiano Mark A, Cheung Alfred K, Weintraub William S, Moran Andrew E, SPRINT Research Group (2017). Cost-effectiveness of intensive versus standard blood-pressure control. N Engl J Med.

[35] Eysenbach Gunther, CONSORT-EHEALTH Group (2011). CONSORT-EHEALTH: improving and standardizing evaluation reports of Web-based and mobile health interventions. J Med Internet Res.

[36] Cross RK, Finkelstein J (2007). Feasibility and acceptance of a home telemanagement system in patients with inflammatory bowel disease: a 6-month pilot study. Dig Dis Sci.

[37] de Jong MJ, Boonen A, van der Meulen-de Jong AE, Romberg-Camps MJ, van Bodegraven AA, Mahmmod N, Markus T, Dijkstra G, Winkens B, van Tubergen A, Masclee A, Jonkers DM, Pierik MJ (2020). Cost-effectiveness of telemedicine-directed specialized vs standard care for patients with inflammatory bowel diseases in a randomized trial. Clin Gastroenterol Hepatol.

[38] Yao J, Fekadu G, Jiang X, You JHS (2022). Telemonitoring for patients with inflammatory bowel disease amid the COVID-19 pandemic-a cost-effectiveness analysis. PLoS One.

[39] Ciencia YT, sociedad DLI 6. 4 Población que usa Internet (en los últimos tres meses).

[40] Gellad ZF, Diamond S, Crockett SD, Cross RK (2023). AGA clinical practice update on telemedicine in gastroenterology: commentary. Gastroenterology.

[41] McCombie A, Walmsley R, Barclay M, Ho C, Langlotz T, Regenbrecht H, Gray A, Visesio N, Inns S, Schultz M (2020). A noninferiority randomized clinical trial of the use of the smartphone-based health applications IBDsmart and IBDoc in the care of inflammatory bowel disease patients. Inflamm Bowel Dis.

[42] Higgins PDR, Schwartz M, Mapili J, Zimmermann EM (2005). Is endoscopy necessary for the measurement of disease activity in ulcerative colitis?. Am J Gastroenterol.

[43] Lin J, Chen J, Zuo J, Yu A, Xiao Z, Deng F, Nie B, Jiang B (2014). Meta-analysis: fecal calprotectin for assessment of inflammatory bowel disease activity. Inflamm Bowel Dis.

[44] Zittan E, Kabakchiev B, Kelly OB, Milgrom R, Nguyen GC, Croitoru K, Steinhart AH, Silverberg MS (2017). Development of the Harvey-Bradshaw Index-pro (HBI-PRO) Score to assess endoscopic disease activity in Crohn's disease. J Crohns Colitis.

[45] Marín-Jiménez Ignacio, Nos P, Domènech Eugeni, Riestra S, Gisbert JP, Calvet X, Cortés Xavier, Iglesias E, Huguet JM, Taxonera C, Fernández Ramón, Carpio D, Gutiérrez Ana, Guardiola J, Laria LC, Sicilia B, Bujanda L, Cea-Calvo L, Romero C, Rincón Óscar, Juliá Berta, Panés Julián (2016). Diagnostic performance of the simple Clinical Colitis Activity Index self-administered online at home by patients with ulcerative colitis: CRONICA-UC study. Am J Gastroenterol.

